# Author Correction: SLC6A20 transporter: a novel regulator of brain glycine homeostasis and NMDAR function

**DOI:** 10.1038/s44321-024-00125-y

**Published:** 2024-09-06

**Authors:** Mihyun Bae, Junyeop Daniel Roh, Youjoung Kim, Seong Soon Kim, Hye Min Han, Esther Yang, Hyojin Kang, Suho Lee, Jin Yong Kim, Ryeonghwa Kang, Hwajin Jung, Taesun Yoo, Hyosang Kim, Doyoun Kim, Heejeong Oh, Sungwook Han, Dayeon Kim, Jinju Han, Yong Chul Bae, Hyun Kim, Sunjoo Ahn, Andrew M Chan, Daeyoup Lee, Jin Woo Kim, Eunjoon Kim

**Affiliations:** 1 Center for Synaptic Brain Dysfunctions, Institute for Basic Science (IBS), Daejeon, 34141 Korea; 2 Department of Biological Sciences, Korea Advanced Institute for Science and Technology (KAIST), Daejeon, 34141 Korea; 3 Therapeutics and Biotechnology Division, Korea Research Institute of Chemical Technology (KRICT), Daejeon, 34114 Korea; 4 Department of Anatomy and Neurobiology, School of Dentistry, Kyungpook National University, Daegu, Korea; 5 Department of Anatomy and Division of Brain Korea 21, Biomedical Science, College of Medicine, Korea University, Seoul, 136-705 Korea; 6 Division of National Supercomputing, KISTI, Daejeon, 34141 Korea; 7 Graduate School of Medical Science and Engineering, KAIST, Daejeon, Korea; 8 School of Biomedical Sciences, Lo Kwee-Seong Integrated Biomedical Sciences Building, The Chinese University of Hong Kong, Hong Kong SAR, China

## Abstract

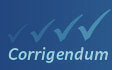

**Correction to:** *EMBO Molecular Medicine* (2021) 13:e12632. 10.15252/emmm.202012632 | Published online 11 January 2021

**An IRB number is corrected in the Material and Methods section**.

The final sentence under the subheading, **‘Human neuron culture’**

is corrected from:

Protocols describing the use of human ESCs were approved in accordance with the ethical requirements and regulations of the Institutional Review Board of KAIST (IRB #KA2018‐61, KH2020‐55).

To: (Changes in bold).

Protocols describing the use of human ESCs were approved in accordance with the ethical requirements and regulations of the Institutional Review Board of KAIST **(IRB #KH2017‐109)**.

