# Supplementary material for: SLC6A20 transporter: a novel regulator of brain glycine homeostasis and NMDAR function
Source: EMBO Mol Med. 2021 Jan 11;13(2):e12632. doi: 10.15252/emmm.202012632 (PMC7863395; doi:10.15252/emmm.202012632)
Supplement: Supplementary file 1 — Appendix [file EMMM-13-e12632-s001.pdf]

Appendix

Table of contents:

Appendix Figure S1 Expression of *Slc6a20* mRNAs in *Pten*-positive cells 1

Appendix Figure S2 Normal social interaction, self-grooming, digging and marble burying behaviors in *Pten*$^{ΔC/ΔC}$ mice 2

Appendix Figure S3 Normal levels of locomotor activity, anxiety-like behavior, and spatial memory in *Pten*$^{ΔC/ΔC}$ mice. 4

Appendix Figure S4 Normal levels of spatial learning, nesting, mother-attachment behavior, and juvenile play in *Pten*$^{ΔC/ΔC}$ mice. 6

Appendix Figure S5 Generation and characterization of *Slc6a20a*-mutant mice 7

Appendix Figure S6 Amino acid sequence alignment of human, mouse, and rat SLC6A20 proteins.. 8

Appendix Figure S7 Examples of current traces from IonFlux auto-patch experiments 10

Appendix Figure S8 SLC6A20A transports sarcosine but not histidine or GABA.............................. .12

Appendix Table S1 Statistics 14

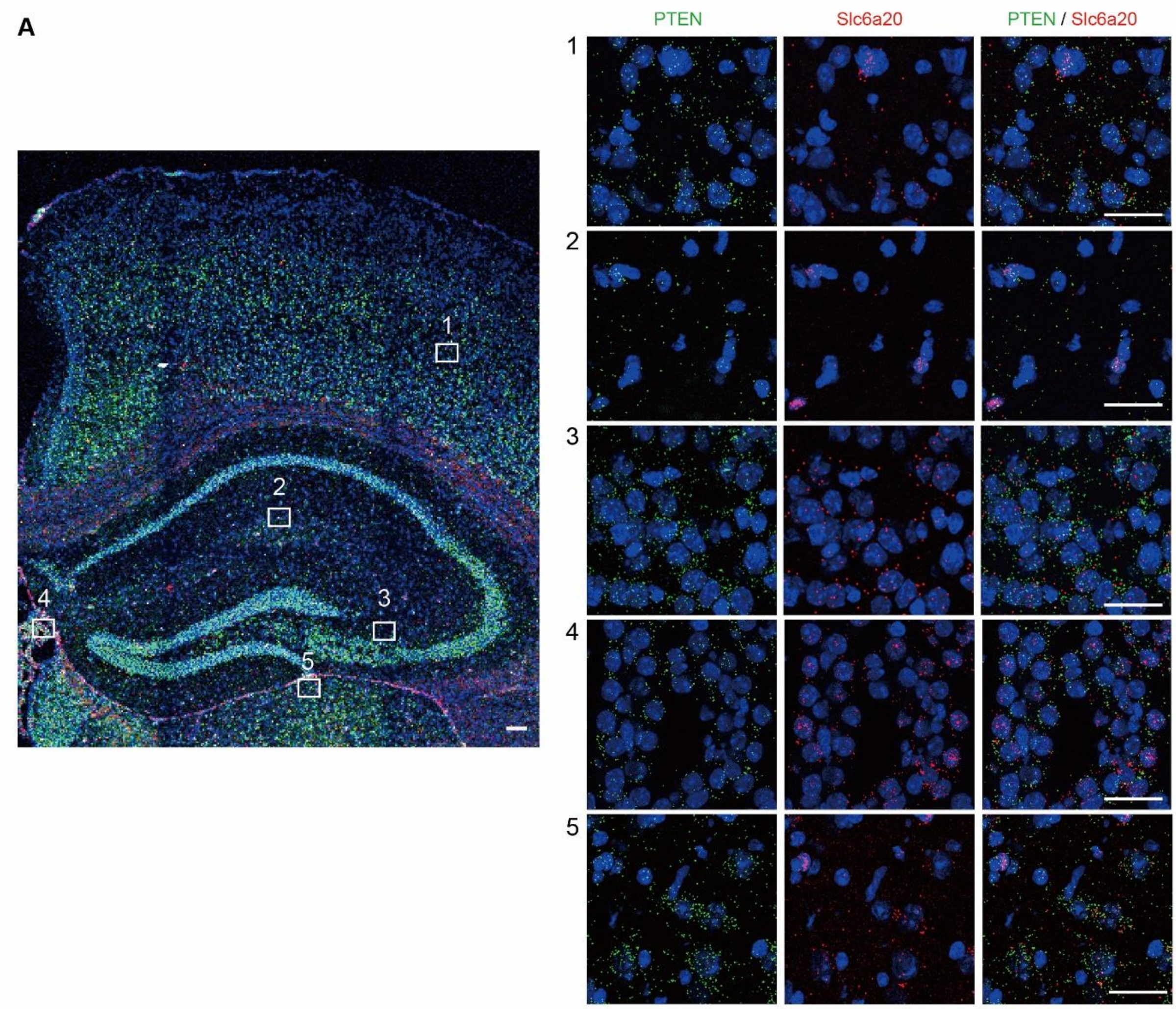

Appendix Figure S1. Expression of *Slc6a20* mRNAs in *Pten*-positive cells.

Expression of *Slc6a20* mRNAs in *Pten*-positive cells in mouse brain regions (P56), including the cortex, hippocampus, and choroid plexus, revealed by double fluorescence in situ hybridization. Note that *Slc6a20* mRNA signals are stronger in the choroid plexus. Scale bar, 100 μm.

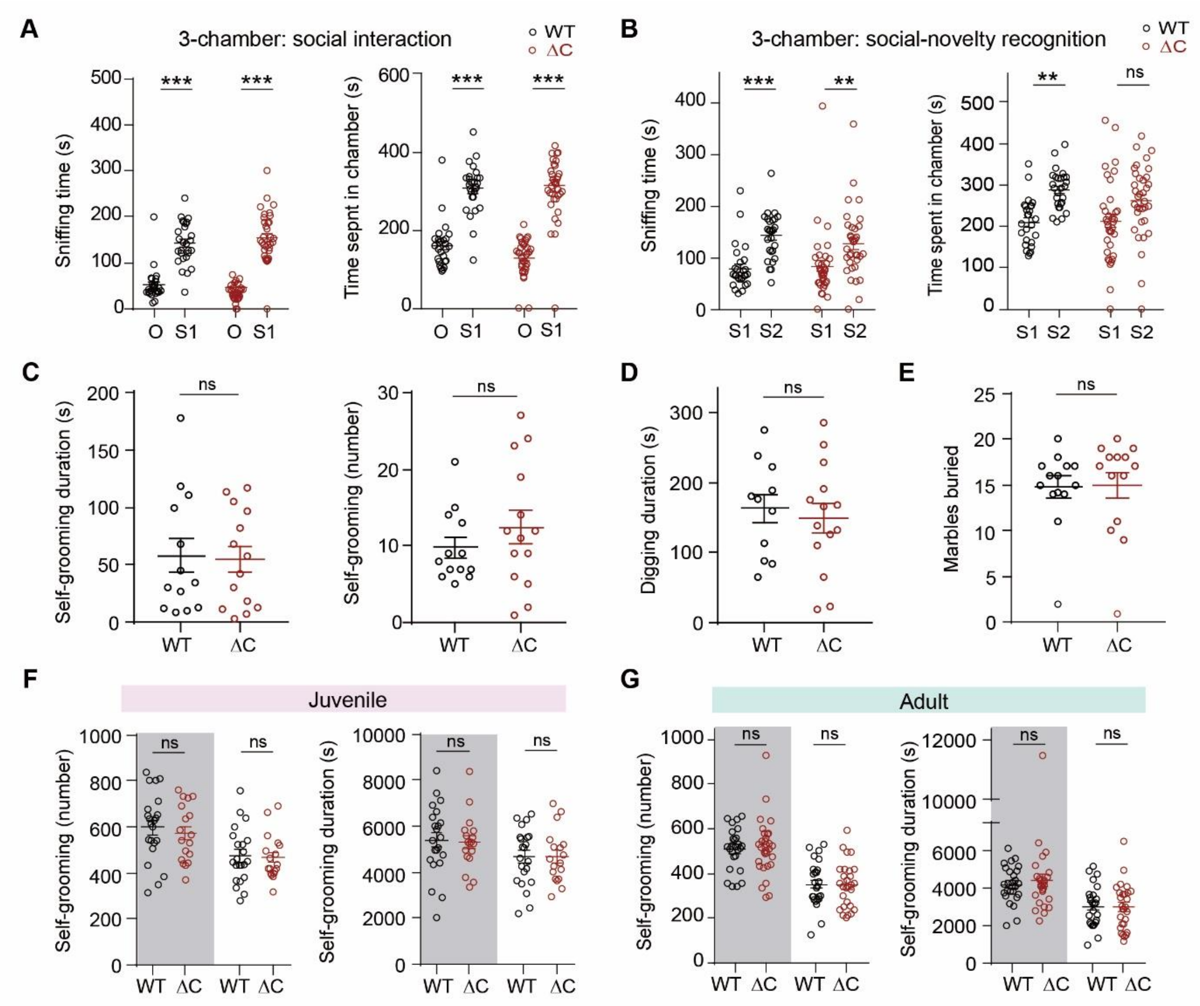

Appendix Figure S2. Normal social interaction, self-grooming, digging and marble burying behaviors in *Pten*$^{\Delta C/\Delta C}$ mice

(A and B) Largely normal levels of social interaction (A) and social-novelty recognition (B) in *Pten*$^{\Delta C/\Delta C}$ mice (2–4 months) in the three-chamber test, as shown by the time spent in sniffing and chamber. S1, stranger mouse; O, inanimate object; S2, a new stranger mouse. (n = 27 mice for WT and 35 for ΔC, $^{**}P < 0.01$, $^{***}P < 0.001$, ns, not significant, repeated measures two-way ANOVA with Bonferroni's test).

(C–E) Normal levels of repetitive behaviors in *Pten*$^{\Delta C/\Delta C}$ mice (1 month for self-grooming and digging; 2–4 months for marble burying) in home cages, as shown by the levels of self-grooming (C), digging (D), and marble burying (E). (n = 13 for WT and 14 for ΔC for self-grooming, 11 for WT and 14 for ΔC for digging, and 13 for WT and 14 for ΔC for marble burying, ns, not significant, Mann-Whitney U test, Student's

t-test).

(F and G) Normal levels of self-grooming in *Pten*$^{\Delta C/\Delta C}$ mice at P30 (F) and 2–4 months (G), as indicated by frequency and time spent self-grooming in Laboras cages, where mouse movements were continuously monitored for 72 hours. Shaded and unshaded periods; 12-hour light-off and light-on periods over 72 hours. (n = 22 mice for WT and 18 mice for ΔC for P30, and 25 for WT and 27 for ΔC for 2–4 months, ns, not significant, Mann-Whitney U test, Student's t-test).

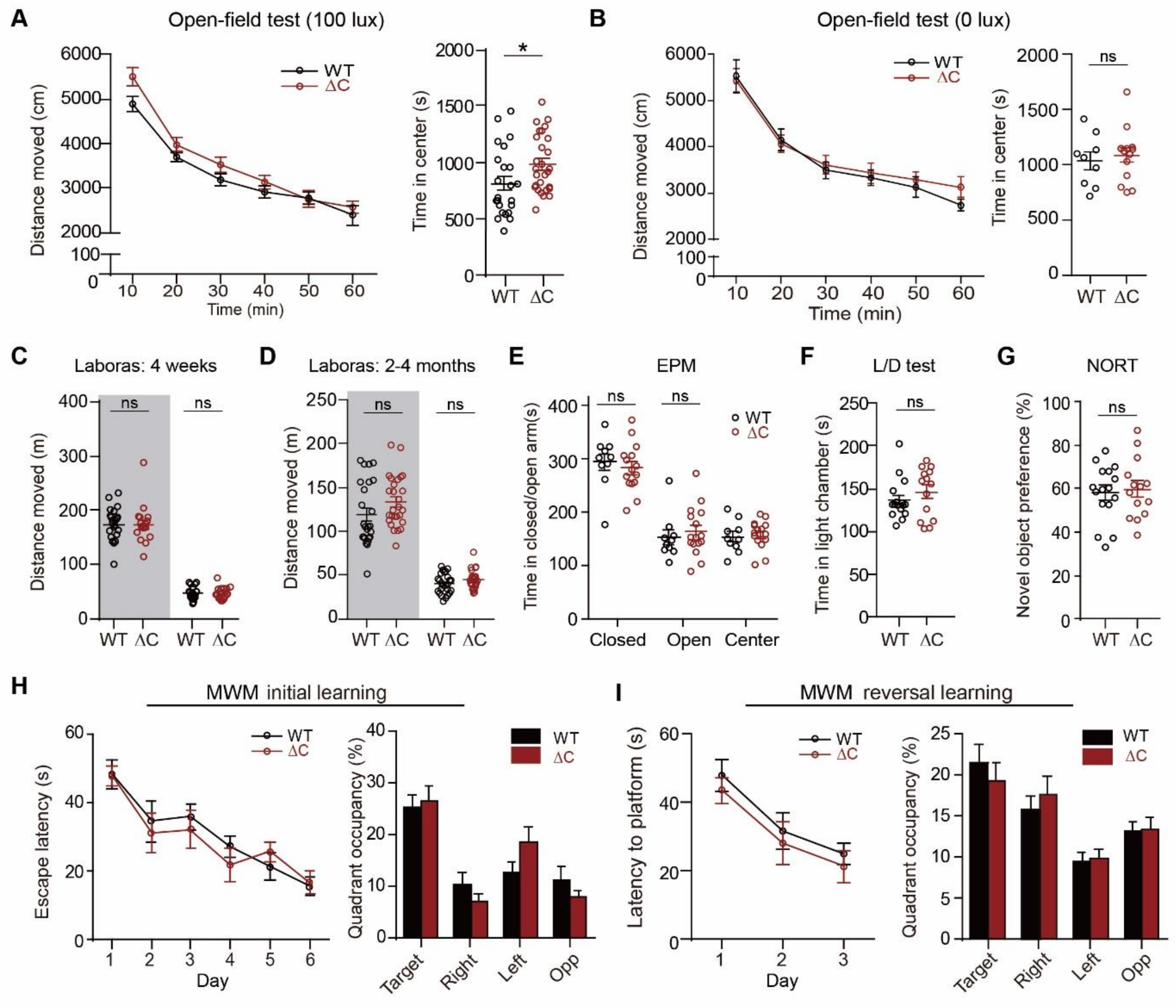

Appendix Figure S3. Normal levels of locomotor activity, anxiety-like behavior, and spatial memory in *Pten*$^{\Delta C/\Delta C}$ mice.

(A and B) Normal locomotor activity in *Pten*$^{\Delta C/\Delta C}$ mice (2–4 months) in the open-field test under conditions of bright light (100 lux; A) and complete darkness (0 lux; B). Note that *Pten*$^{\Delta C/\Delta C}$ mice spent increased amounts of time spent in the center region of the open-field arena. (n = 23 mice for WT and 28 for ΔC for bright-light and 10 for WT and 14 for ΔC for complete-darkness, *P < 0.05, ns, not significant, repeated measures of two-way ANOVA with Bonferroni's test, Mann-Whitney U test).

(C and D) Normal locomotor activity in *Pten*$^{\Delta C/\Delta C}$ mice at 4 weeks (C) and 2–4 months (D) in Laboras cages, where mouse movements were continuously measured for 69 hrs (adult) and 72 hrs (juvenile). (n = 22 mice for WT and 18 mice for ΔC for 4 weeks, and 25 for WT and 27 for ΔC for 2-4 months, ns, not significant, Mann- Whitney U test, Student's t-test).

(E) Normal anxiety-like behavior in *Pten*$^{\Delta C/\Delta C}$ mice (2–4 months) in the elevated plus-maze test. (n = 10 mice for WT and 15 for ΔC, ns, not significant, Mann-Whitney U test, Student’s t-test).

(F) Normal anxiety-like behavior in *Pten*$^{\Delta C/\Delta C}$ mice (2–4 months) in the light-dark test. (n = 16 mice for WT and 14 for ΔC, ns, not significant, Mann-Whitney U test).

(G) Normal novel object recognition in *Pten*$^{\Delta C/\Delta C}$ mice (2–4 months). (n= 16 mice for WT and 14 for ΔC, ns, not significant, Student’s t-test).

(H and I) Normal levels of spatial learning and memory in *Pten*$^{\Delta C/\Delta C}$ mice (2–4 months) in the learning (H, left), probe (H, right), reversal (I, left), and probe (I, right) phases of the Morris water-maze test. Opp, opposite. (n = 8 mice for WT and 8 for ΔC, ns, not significant, repeated measures of two-way ANOVA with Bonferroni’s test for escape latency; Mann-Whitney U test and Student’s t-test for quadrant occupancy).

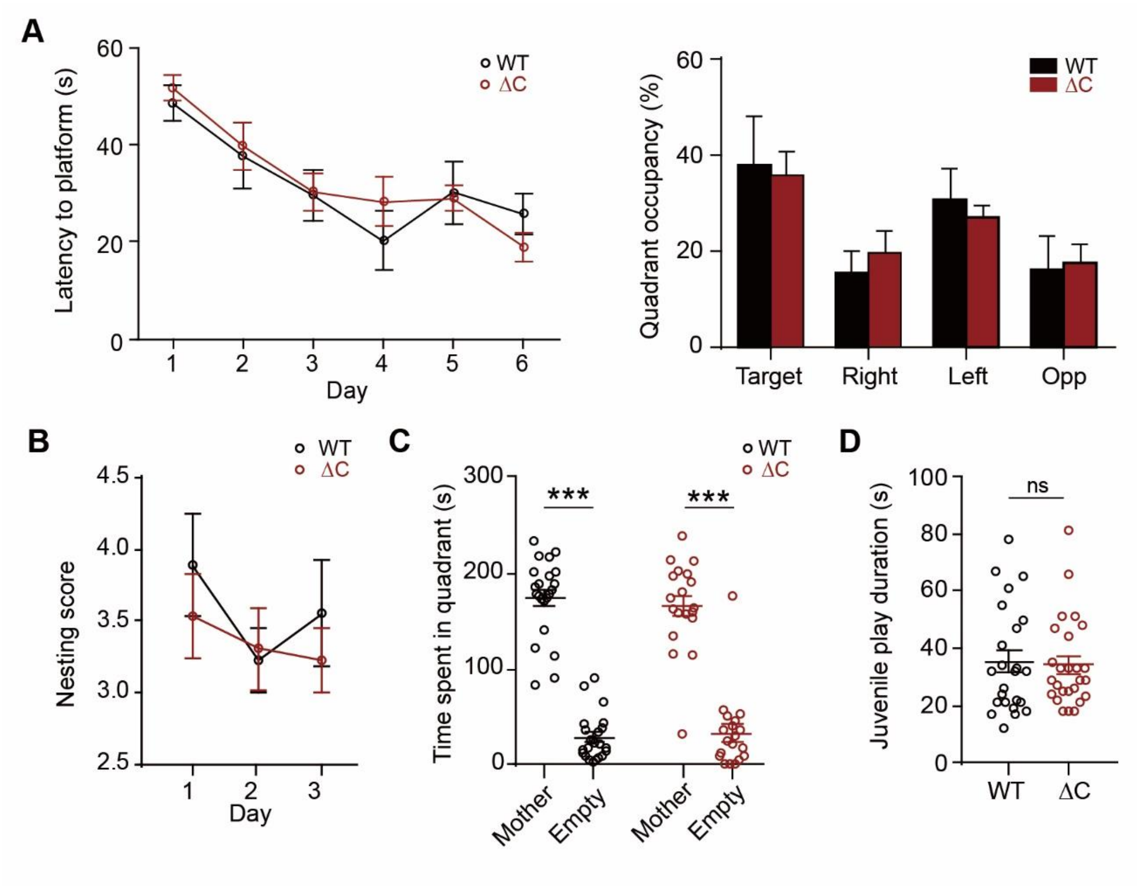

Appendix Figure S4. Normal levels of spatial learning, nesting, mother-attachment behavior, and juvenile play in *Pten*$^{\Delta C/\Delta C}$ mice.

(A) Normal spatial learning and long-term (7-day) memory in *Pten*$^{\Delta C/\Delta C}$ mice (2–4 months) in the learning (left) and probe (right; 7 days later) phases of the Morris water-maze test. Opp, opposite. (n = 7 mice for WT and 9 for ΔC, ns, not significant, repeated measures of two-way ANOVA with Bonferroni's test for escape latency; Mann-Whitney U test and Student's t-test for quadrant occupancy).

(B) Normal nest building in *Pten*$^{\Delta C/\Delta C}$ mice (2–4 months), as shown by the nesting scores over 3 days. (n = 9 mice for WT and 13 mice for ΔC, ns, not significant, Student's t-test).

(C) Normal mother-seeking behavior in *Pten*$^{\Delta C/\Delta C}$ mice (3 weeks) in the maternal homing test, as shown by time spent by a juvenile mouse with the reunited mother. (n = 22 mice for WT and 19 mice ΔC, ***P < 0.001, repeated measures of two-way ANOVA with Bonferroni's test).

(D) Normal juvenile play in *Pten*$^{\Delta C/\Delta C}$ mice (3 weeks), as shown by the time spent in juvenile play. (n = 24 mice for WT and 26 mice for ΔC, ns, not significant, Mann-Whitney U test).

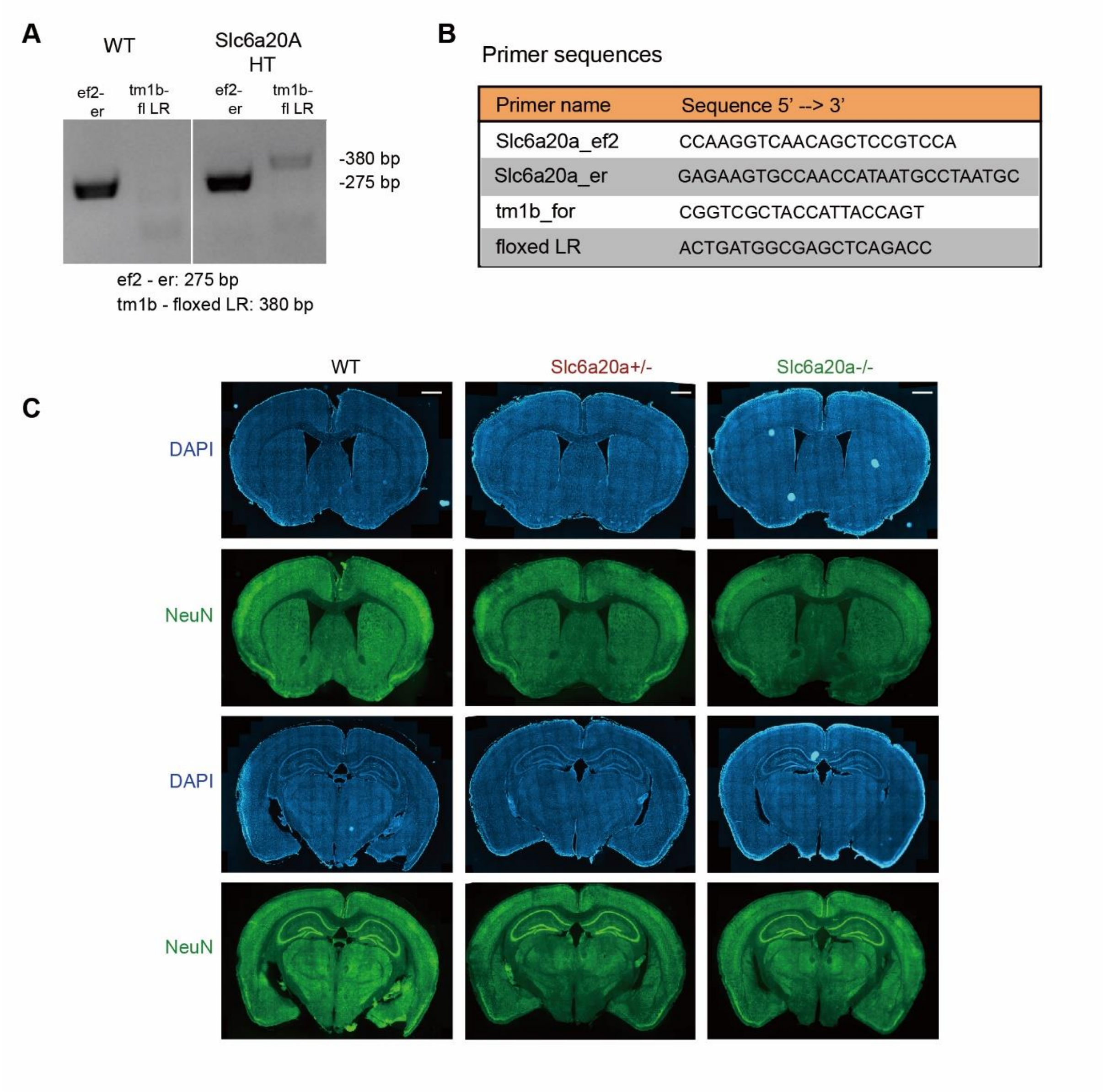

Appendix Figure S5. Generation and characterization of *Slc6a20a*-mutant mice.

(A) PCR genotyping of *Slc6a20a*-mutant mice.

(B) Nucleotide sequences of the primers used for PCR genotyping.

(C) Largely normal gross morphology of the brain in heterozygous *Slc6a20a*$^{+/-}$ and homozygous *Slc6a20a*$^{-/-}$ mice, as indicated by immunofluorescence staining for NeuN (neuronal marker) and DAPI (nuclear marker for all types of cells). Scale bar, 100 µm.

A

```
human SLC6A20 v1    -------------------------------------------MEKARPLWANSLQFVFA 17
human SLC6A20 v2    -------------------------------------------MEKARPLWANSLQFVFA 17
mouse slc6a20 A     -------------------------------------------MEKARPQWGHPLQFVFA 17
mouse slc6a20 B     MESPSAHAVSLPEDEELQPWGGAGGPGQHPGRPRSTECAHPGVVEKVRPKWDNPLQFLLV 60
Rat slc6a20         ---------MRL-------AIKRRASRGQRPGP----DEKRARDMEKARPQWGNPLQFVFA 41
                                                                     ** ** *   ***

human SLC6A20 v1    CISYAVGLGNVWRFPYLCQMYGGGSFLVPYIIMLIVEGMPLLYLELAVGQRMRQGSIGAW 77
human SLC6A20 v2    CISYAVGLGNVWRFPYLCQMYGGGSFLVPYIIMLIVEGMPLLYLELAVGQRMRQGSIGAW 77
mouse slc6a20 A     CISYAVGLGNVWRFPYLCQMYGGGSFLVPYIIMLIVEGMPLLYLELAVGQRMRQGSIGAW 77
mouse slc6a20 B     CISYAVGLGNVWRFPYLCQMYGGGNFLVPYIIMLIVEGMPLLYLELAVGQRMRQGSIGAW 120
Rat slc6a20         CISYAVGLGNVWRFPYLCQMYGGGSFLVPYLIMLIVEGMPLLYLELAVGQRMRQGSIGAW 101
                    ************************ ***** *****************************

human SLC6A20 v1    RTISPYLSGVGVASVVVSFFLSMYYNVINAWAFWYLFHSFQDPLPWSVCPLNGNHTGYDE 137
human SLC6A20 v2    RTISPYLSGVGVASVVVSFFLSMYYNVINAWAFWYLFHSFQDPLPWSVCPLNGNHTGYDE 137
mouse slc6a20 A     RTISPYLSGVGVASVVVSFFLSMYYNVINAWGFWYLFHSFQDPLPWSVCPLNSNHTGYDE 137
mouse slc6a20 B     RTISPYLSGVGIASLVVSFLASVYFNVINTWALWYLFHSFQDPLPWSVCPLNSNHTGYDE 180
Rat slc6a20         RTISPYLSGVGVASVVVSFFLSMYYNVINAWGFWYLFHSFQDPLPWSVCPLNSNRTGYDE 161
                    *********** ** ****  * * **** *  ******************* * *****

human SLC6A20 v1    ECEKASSTQYFWYRKTLNISPSLQENGGVQWEPALCLLLAWLVVYLCILRGTESTGKVVY 197
human SLC6A20 v2    ECEKASSTQYFWYRKTLNISPSLQENGGVQWEPALCLLLAWLVVYLCILRGTESTGK--- 194
mouse slc6a20 A     ECEKASSTQYFWYRKTLNISPSIQENGGVQWEPALCLTLAWLMVYLCILRGTESTGKVVY 197
mouse slc6a20 B     ECEKASSTQYFWYRKTLNISPSIQENGGVQWEPALCLTLAWLMVYLCILRGTESTGKVVY 240
Rat slc6a20         ECEKASSTQYFWYRKTLNISPSIQENGGVQWEPALCLTLAWLMVYLCILRGTESTGKVVY 221
                    ********************** ************** **** **************

human SLC6A20 v1    FTASLPYCVLIIYLIRGLTLHGATNGLMYMFTPKIEQLANPKAWINAATQIFFSLGLGFG 257
human SLC6A20 v2    ----------------------------------IEQLANPKAWINAATQIFFSLGLGFG 220
mouse slc6a20 A     FTASMPYCVLIIYLVRGLTLHGATNGLMYMFTPKMEQLANPKAWINAATQIFFSLGLGFG 257
mouse slc6a20 B     FTTSLPYFVLIIYLVRGLTLHGATNGLAYMFTPKIEQLANPKAWINAATQIFFSLGLCG 300
Rat slc6a20         FTALMPYCVLIIYLVRGLTLHGATNGLMYMFTPKIEQLANPKAWINAATQIFFSLGLGFG 281
                    ##   ## ###### ############ ###### *********************** *

human SLC6A20 v1    SLIAFASYNEPSNNCQKHAIIVSLINSFTSIFASIVTFSIYGFKATFNYENCLKKVSLLL 317
human SLC6A20 v2    SLIAFASYNEPSNNCQKHAIIVSLINSFTSIFASIVTFSIYGFKATFNYENCLKKVSLLL 280
mouse slc6a20 A     SLIAFASYNEPSNNCQKHAIIVSIINSSTSIFASIVTFSIYGFKATFNYENCLNKVILLL 317
mouse slc6a20 B     GLIAFASYNEPSNDCQKHALIVSVINSTTAIFSSIVTFSIYGFKATFNYENCLNKVILLL 360
Rat slc6a20         SLIAFASYNEPSNDCQKHAVIVSVINSSTSIFASIVTFSIYGFKATFNYENCLNKVILLL 341
                     ************ ***** *** *** * ** ******************** ** ***

human SLC6A20 v1    TNTFDLEDGFLTASNLEQVKGYLASAYPSKYSEMFPQIKNCSLESELDTAVQGTGLAFIV 377
human SLC6A20 v2    TNTFDLEDGFLTASNLEQVKGYLASAYPSKYSEMFPQIKNCSLESELDTAVQGTGLAFIV 340
mouse slc6a20 A     TNSFDLEDGFLTVSNLEEVKNYLASTYPNKYSEVFPHIRNCSLESELDTAVQGTGLAFIV 377
mouse slc6a20 B     TNSFDLEDGFLTVSNLEEVKNYLASTYPNKYSEVFPHIRNCSLESELDTAVQGTGLAFIV 420
Rat slc6a20         TNSFDLEDGFLTASNLEEVKDYLASTYPNKYSEVFPHIRNCSLESELNTAVQGTGLAFIV 401
                    ** ********* **** ** **** ** **** ** * ******** ************

human SLC6A20 v1    YTEAIKNMEVSQLWSVLYFFMLLMLGIGSMLGNTAAILTPLTDSKIISSHLPKEAISGLV 437
human SLC6A20 v2    YTEAIKNMEVSQLWSVLYFFMLLMLGIGSMLGNTAAILTPLTDSKIISSHLPKEAISGLV 400
mouse slc6a20 A     YTEAIKNMEVSQLWSVLYFFMLLMLGIGSMLGNTAAILTPLTDSKVISSYLPKEAISGLV 437
mouse slc6a20 B     YTEAIKNMEVSQLWSVLYFFMLLTLGMGSMVGTGTAILTPLTDSKIISSYLPKEAISGLV 480
Rat slc6a20         YAEAIKNMEVSQLWSVLYFFMLLMLGMGSMLGNTAAILTPLTDSKVISSYLPKEAISGLV 461
                    * ********************* ** *** *   ********** *** **********

human SLC6A20 v1    CLVNCAIGMVFTMEAGNYWFDIFNDYAATLSLLLIVLVETIAVCYVYGLRRFESDLKAMT 497
human SLC6A20 v2    CLVNCAIGMVFTMEAGNYWFDIFNDYAATLSLLLIVLVETIAVCYVYGLRRFESDLKAMT 460
mouse slc6a20 A     CLINCAVGMVFTMEAGNYWFDIFNDYAATLSLLLIVLVETIAVCYVYGLKRFESDLRAMT 497
mouse slc6a20 B     CLLNCAIGMVFTMEAGNYWFDLFNDYTATLSLLLIVLVETIAVCYVYGLKRFESDLRAMT 540
Rat slc6a20         CLINCAVGMVFTMEAGNYWFDIFNDYAATLSLLLIVLVETIAVCYVYGLRRFESDLRAMT 521
                    ** *** ************** **** ********************** ****** ***

human SLC6A20 v1    GRAVSWYWKVMWAGVSPLLIVSLFVFYLSDYILTGTLKYQAWDASQGQLVTKDYPAYALA 557
human SLC6A20 v2    GRAVSWYWKVMWAGVSPLLIVSLFVFYLSDYILTGTLKYQAWDASQGQLVTKDYPAYALA 520
mouse slc6a20 A     GRTLSWYWKVMWAFVSPLLIVGLFIFYLSDYILTGTLQYQAWDATQGQLVTKDYPPHALA 557
mouse slc6a20 B     GRTLSWYWKVMWAFVSPLLIVGLFIFYLSDYILTGTLQYQAWDATQGHVVTKDYPTYALA 600
Rat slc6a20         GRPLNWYWKAMWAFVSPLLIIGLFIFYLSDYILTGTLQYQAWDATQGQLVTKDYPPHALA 581
                    **   ****  *** ******  ** ************ ****** **  ******  ***

human SLC6A20 v1    VIGLLVASSTMCIPLAALGTFVQRRLKRGDADPVA 592
human SLC6A20 v2    VIGLLVASSTMCIPLAALGTFVQRRLKRGDADPVA 555
mouse slc6a20 A     VIGLLVASSTMCIPLVALGTFIRNRLKRGGSAPVA 592
mouse slc6a20 B     VIGLLVASSTMCIPLVALGTFVTRHFKIREQFSAA 635
Rat slc6a20         VIGLLVASSTMCIPLVALGTFIRNRLKRGGSSPVA 616
                    *************** *****     *       *
```

Appendix Figure S6. Amino acid sequence alignment of human, mouse, and rat SLC6A20 proteins.

(A) Amino acid sequence alignment of human and mouse SLC6A20 proteins used for proline/glycine transports. v1 and v2, two different splice variants of human SLC6A20 (Genbank numbers aa sequences: NP_064593 and NP_071800, respectively). SLC6A20 A and B, two different SLC6A20 proteins encoded by two independent *Slc6a20* genes (*Slc6a20a* and *Slc6a20b*) in mice (Genbank numbers for aa sequences: NP_631881 and NP_035861, respectively). Rat SLC6A20 (Genebank number: NP_579830).

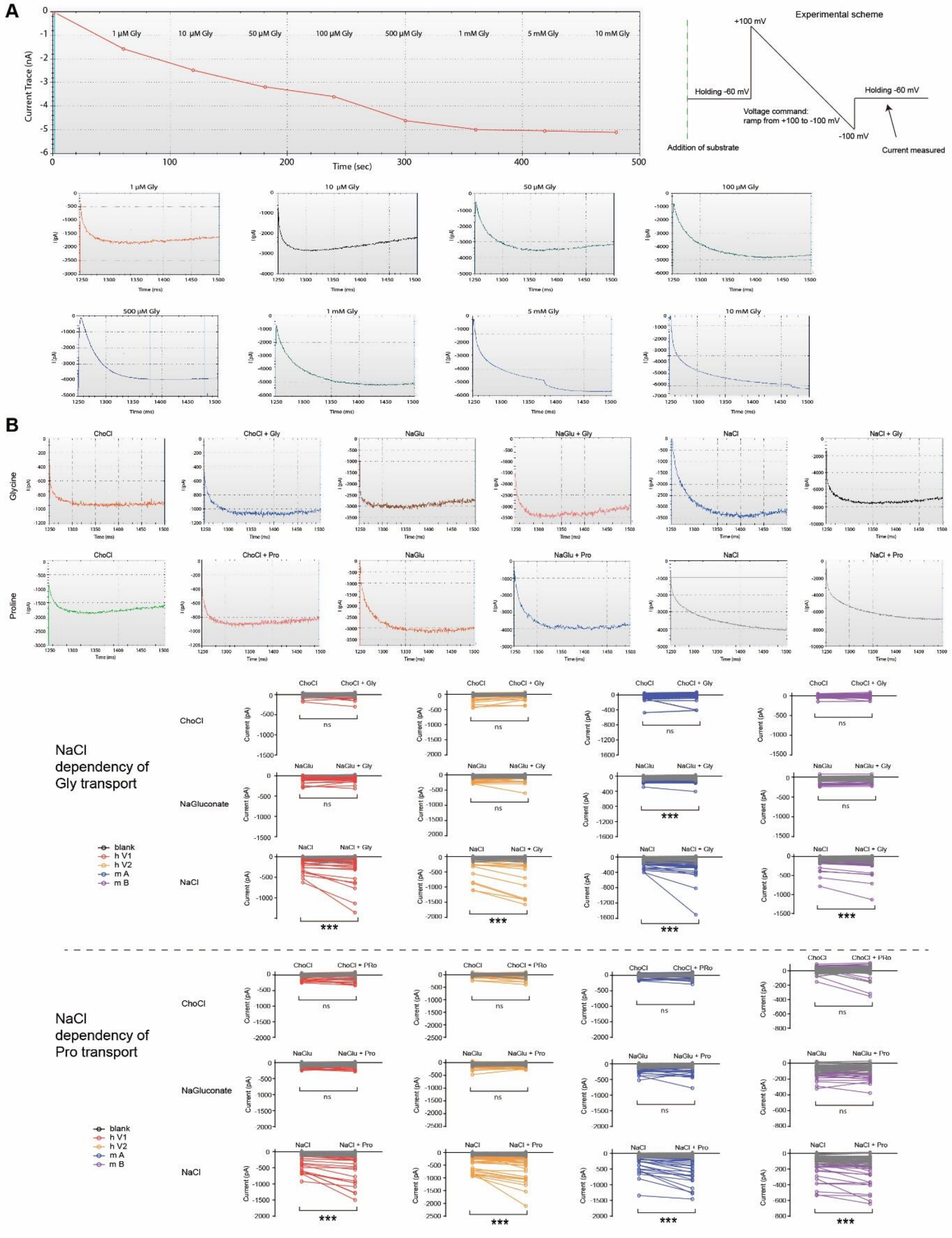

Appendix Figure S7 Examples of current traces from IonFlux auto-patch experiments.

(A) (top left) An example of the experimental schemes in IonFlux auto-patch experiments where glycine/proline-evoked currents were measured from HEK293T

cells expressing SLC6A20 variants stimulated sequentially with increasing concentrations of glycine/proline with 60-sec intervals (0, 1, 10, 50, 100, 500, 1,000, 5,000, 10,000 μM). The example shown is for glycine-evoked currents for mSCL6A20A.

(top right) HEK293T cells expressing SLC6A20 protein variants in 100 mM NaCl medium (pH 7.5) were initially clamped at -60 mV, followed the ramps of membrane potentials (Vm) ranging from +100 to -100 mV druing ~1 sec after the addition of substrates, re-clamping at -60 mV, and measurements of evoked currents.

(bottom) Examples of current traces from HEK293T cells expressing mSLC6A20A evoked by sequential increases of glycine concentrations (0, 1, 10, 50, 100, 500, 1,000, 5,000, 10,000 μM) and observed immediately after the re-clamping at -60 mV.

(B) (top) Examples of current traces from IonFlux auto-patch experiments where HEK293T cells expressing mSLC6A20A were evoked by sequential changes of experimental conditions (i.e. presence of choline chloride [ChoCl], sodium gluconate [NaGlu], or sodium chloride [NaCl]) + presence/absence of glycine/proline) at the holding potential of –60 mV.

(bottom) Additional details of NaCl dependency of the glycine/proline-evoked currents of SLC6A20 variants (human and mouse) summarized in **Fig. 7B**. HEK293T cells expressing SLC6A20 variants were evoked by sequential changes of experimental conditions (i.e. presence/absence of glycine/proline under choline chloride, sodium gluconate, or sodium chloride (NaCl) conditions). (glycine, n = 21 cells for untransfected/blank, 17 for human SLC6A20-V1, 25 for human SLC6A20-V2, 33 for mSLC6A20A, 28 for mSLC6A20B ***P < 0.001 (relative to buffer not containing glycine), two-way ANOVA with Bonferroni's test; proline, n = 17 cells for untransfected, 23 for human SLC6A20-V1, 47 for SLC6A20-V2, 31 for mSLC6A20A, and 31 for mSLC6A20B, ***P < 0.001, two-way ANOVA with Bonferroni's test).

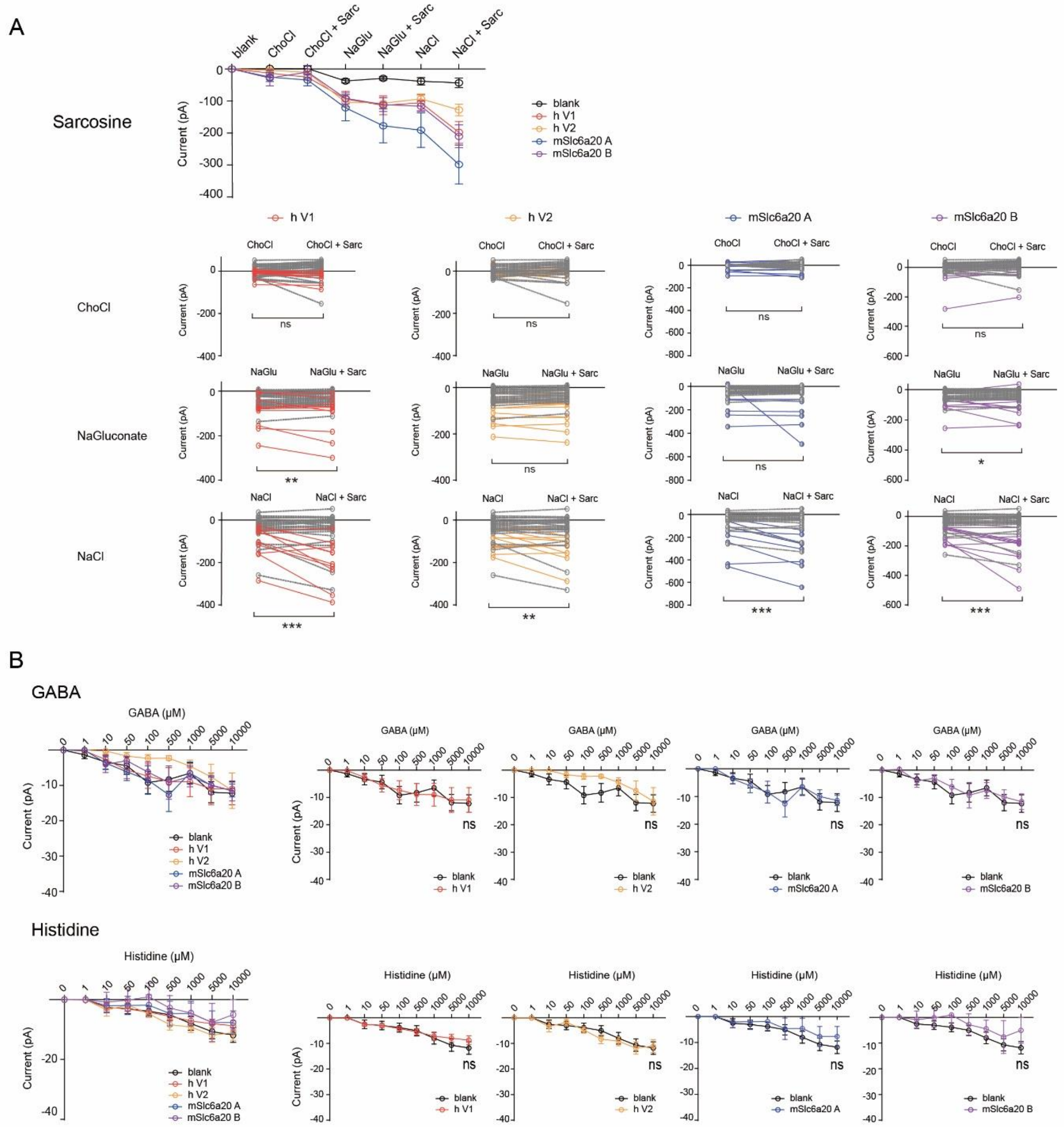

Appendix Figure S8. SLC6A20A transports sarcosine but not histidine or GABA.

(A) SLC6A20A transports sarcosine (a positive control substrate) in a sodium chloride-dependent manner in HEK293T cells expressing mouse SLC6A20A, as shown by peak currents elicited by the indicated increasing concentrations of sarcosine sequentially added in auto-patch experiments. Sodium chloride was replaced with choline chloride (ChoCl) or sodium gluconate (NaGlu) to determine the dependence of the transport on sodium chloride. The datasets in the first graph were separated to subsets in the following graphs for better visibility. (n = 27 cells for

untransfected/blank HEK293T cells, 10 for human SLC6A20-V1, 12 for human SLC6A20-V2, 9 for mouse SLC6A20A, 12 for mouse SLC6A20B, *P < 0.05, **P < 0.01, ***P < 0.001, ns, not significant [relative to the currents without substrates], two-way ANOVA with Bonferroni's test).

(B) SLC6A20A does not transport histidine or GABA (negative control substrates), as shown by peak currents elicited by the indicated concentrations of histidine/GABA in auto-patch experiments. The datasets in the first graph were separated to subsets in the following graphs for better visibility. (For histidine, n = 8 cells for non-transfected HEK293T cells, 11 for human SLC6A20-v1, 10 for human SLC6A20-v2, 11 for mouse SLC6A20A, 11 for mouse SLC6A20B, two-way ANOVA with Bonferroni's test; for GABA, n = 9 cells for non-transfected HEK293T cells, 16 for human SLC6A20-v1, 8 for human SLC6A20-v2, 7 for mouse SLC6A20A, 11 for mouse SLC6A20B, ns, not significant [relative to untransfected/blank], two-way ANOVA with Bonferroni's test).
