# Supplementary material for: SLC6A20 transporter: a novel regulator of brain glycine homeostasis and NMDAR function
Source: EMBO Mol Med. 2021 Jan 11;13(2):e12632. doi: 10.15252/emmm.202012632 (PMC7863395; doi:10.15252/emmm.202012632)
Supplement: Supplementary file 2 — Expanded View Figures PDF [file EMMM-13-e12632-s002.pdf]

Expanded View Figures

Figure EV1. Characterization of *Pten*$^{\Delta C/\Delta C}$ mice.

A Schematic depiction of the generation of *Pten*$^{\Delta C/\Delta C}$ (ΔC) mice, and PCR genotyping of WT, *Pten*$^{\Delta C/\Delta C}$ and *Pten*$^{\Delta C/+}$ (denoted as WT, ΔC, ΔC/+) mice.

B, C Normal gross morphology of the brain in *Pten*$^{\Delta C/\Delta C}$ mice (P21), as revealed by staining for NeuN (a neuronal maker) in coronal sections. Scale bar, 1 mm.

D–F Immunoblot analysis of the levels of total PTEN and phospho-PTEN (pPTEN) proteins in whole-brain total lysates (D; P21), whole-brain crude synaptosomes (E, P21), and hippocampal crude synaptosomes (F, P21). Average values and ΔC/WT ratios were calculated using PTEN/pPTEN signals in WT and *Pten*$^{\Delta C/\Delta C}$ mice normalized to those of α-tubulin. (n = 4 mice for WT and 3 for ΔC for whole-brain lysates (D), 4 for both WT and ΔC for whole-brain crude synaptosomes (E), and WT 5 and ΔC 8 for hippocampus lysates (F); ns, not significant, Student's t-test). See Appendix Fig S7 for full-length blot images. The error bars represent SEM.

G Total and phosphorylation levels of AKT and mTOR. Average values and ΔC/WT ratios were obtained from signals normalized to β-actin. (n = 4 mice for WT and ΔC, ns, not significant, Student's t-test). The error bars represent SEM.

H, I Levels of synaptic glutamate receptors (GluNs, GluAs, and mGluRs) and synaptic plasticity-related signaling proteins (total/phosphorylated GluA1-Ser831, GluA1-Ser845, GSK3β, ERK1/2, and p38) in the brain (whole-brain crude synaptosomes) of *Pten*$^{\Delta C/\Delta C}$ and WT mice (P21). Average values and ΔC/WT ratios were obtained from signals normalized to α-tubulin. (n = 4 mice for WT and ΔC, ns, not significant, Student's t-test). The error bars represent SEM.

J RT–qPCR validation of the nine DEGs in *Pten*$^{\Delta C/\Delta C}$ and WT mice (P21). Note that eight of the nine DEGs could be validated (n = 3 mice for WT and ΔC, *$P < 0.05$, **$P < 0.01$, ns, not significant, Student's t-test). The error bars represent SEM.

Source data are available online for this figure.

 © 2021 The Authors

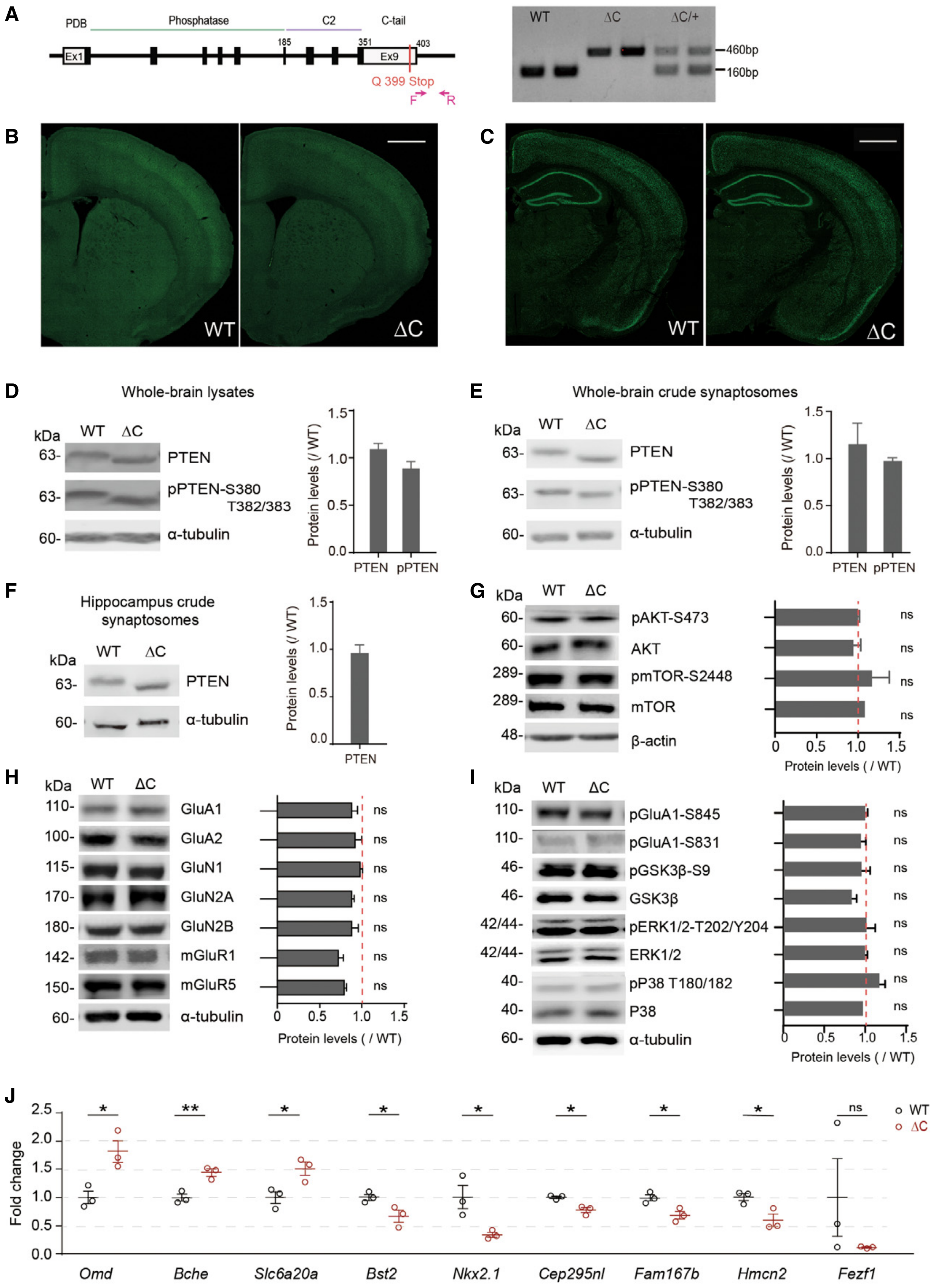
A
PDB
Phosphatase
C2
C-tail
Ex1
185
351
403
Ex9
Q 399 Stop
F
R
WT
ΔC
ΔC/+
460bp
160bp
B
C
WT
ΔC
D
Whole-brain lysates
kDa
63-
60-
PTEN
pPTEN-S380 T382/383
α-tubulin
Protein levels (/ WT)
pPTEN
E
Whole-brain crude synaptosomes
F
Hippocampus crude synaptosomes
G
pAKT-S473
AKT
pmTOR-S2448
mTOR
β-actin
289-
48-
ns
Protein levels (/ WT)
H
GluA1
GluA2
GluN1
GluN2A
GluN2B
mGluR1
mGluR5
I
pGluA1-S845
pGluA1-S831
pGSK3β-S9
GSK3β
pERK1/2-T202/Y204
ERK1/2
pP38 T180/182
P38
J
Fold change
Omd
Bche
Slc6a20a
Bst2
Nkx2.1
Cep295nl
Fam167b
Hmcn2
Fezf1

Figure EV1.

Figure EV2. Reduced mEPSC frequency, increased mIPSCs frequency, and reduced density, but normal morphology, of the PSD in hippocampal DG and CA1 regions in *Pten*$^{\Delta C/\Delta C}$ mice.

A, B Reduced frequency of mEPSCs (A) and increased frequency of mIPSCs (B) in CA1 pyramidal cells in the hippocampus of *Pten*$^{\Delta C/\Delta C}$ mice (P17–21 for mEPSCs; P18–21 for mIPSCs). Data represent mean + SEM (mEPSC, n = 15 neurons from three mice for WT and 14 (3) for ΔC; mIPSC, 13 (4) for WT, and 14 (4) for ΔC; *P < 0.05, ns, not significant, Student's t-test and Mann–Whitney U-test; See Appendix Table S1 for details).

C, D Reduced frequency of mEPSCs (C) and increased frequency of mIPSCs (D) in DG granule cells in the hippocampus of *Pten*$^{\Delta C/\Delta C}$ mice (P18–19 for mEPSCs; P18–23 for mIPSCs). (mEPSC, n = 17 (3) for WT and 14 (3) for ΔC; mIPSC, 23 (5) for WT and 19 (5) for ΔC; *P < 0.05, **P < 0.01, ns, not significant, Student's t-test and Mann–Whitney U-test). The error bars represent SEM.

E, F Reduced frequency of sEPSCs (E), but normal sIPSCs (F), in CA1 pyramidal neurons in the hippocampus of *Pten*$^{\Delta C/\Delta C}$ mice (P17–19 for sEPSCs; P17–21 for sIPSCs). (sEPSC, n = 19 (4) for WT and 17 (4) for ΔC; sIPSC, 20 (4) for WT and 19 (4) for ΔC; *P < 0.05, ns, not significant, Student's t-test and Mann–Whitney U-test). The error bars represent SEM.

G, H Decreased density of excitatory synapses in the hippocampal CA1 and DG regions of *Pten*$^{\Delta C/\Delta C}$ mice. (G), CA1 stratum radiatum; (H), DG molecular layer. Excitatory synapses are defined by sites where PSDs are in contact with presynaptic vesicles. Normal and perforated PSDs are indicated by arrows and arrowheads, respectively. (n = 3 mice for WT and ΔC, ***P < 0.001, ns, not significant, Student's t-test). Scale bar, 200 nm. The error bars represent SEM.

© 2021 The Authors

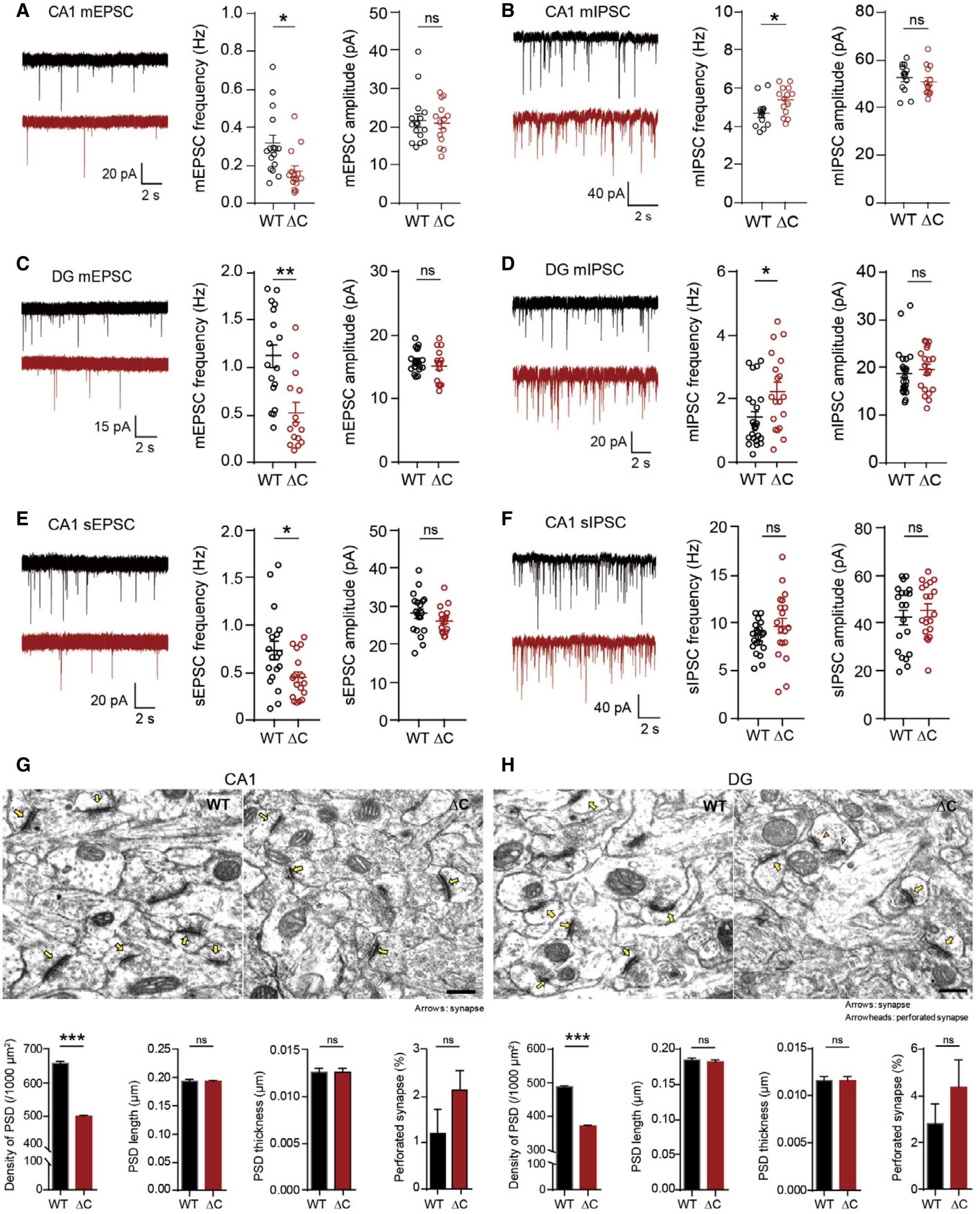
A
CA1 mEPSC
20 pA
2 s
mEPSC frequency (Hz)
mEPSC amplitude (pA)
WT ΔC
B
CA1 mIPSC
40 pA
2 s
mIPSC frequency (Hz)
mIPSC amplitude (pA)
C
DG mEPSC
15 pA
2 s
D
DG mIPSC
20 pA
2 s
E
CA1 sEPSC
20 pA
2 s
sEPSC frequency (Hz)
sEPSC amplitude (pA)
F
CA1 sIPSC
40 pA
2 s
sIPSC frequency (Hz)
sIPSC amplitude (pA)
G
CA1
H
DG
WT
ΔC
Arrows : synapse
Arrowheads : perforated synapse
Density of PSD (/1000 μm²)
PSD length (μm)
PSD thickness (μm)
Perforated synapse (%)
*
**
***
ns

Figure EV2.

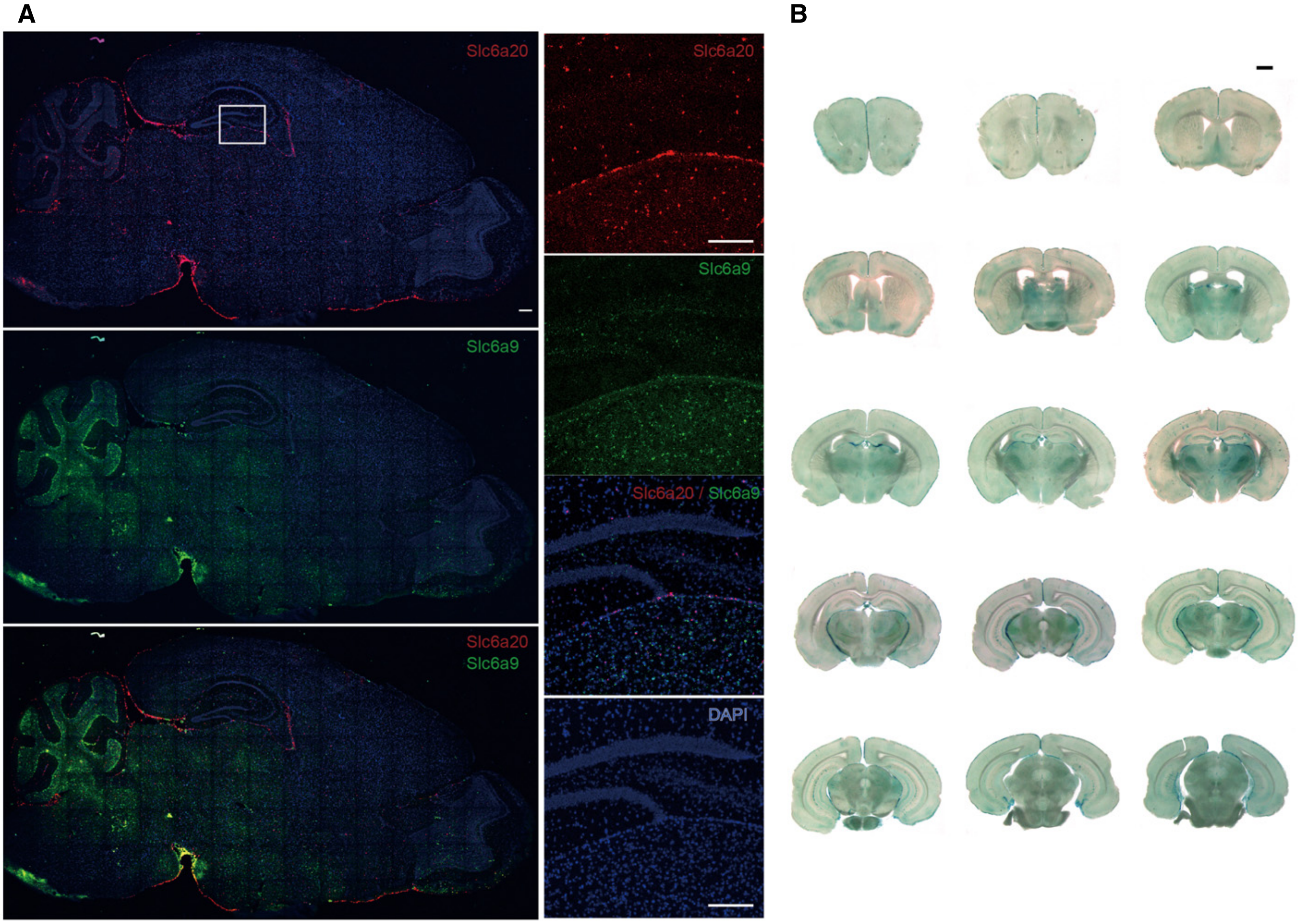

Figure EV3. Widespread expression of Slc6a20 mRNA and SLC6A20A β-gal fusion proteins in brain regions, including the meninges.

A Widespread expression of *Slc6a20* mRNA in various mouse brain regions (P56), including the meninges, cortex, hippocampus, choroid plexus, and thalamus, revealed by double fluorescence *in situ* hybridization (FISH). *Slc6a9* mRNA, encoding GlyT1, was also detected together with *Slc6a20* mRNA for comparison. Note that *Slc6a20* mRNA shows partial colocalization with *Slc6a9* mRNA. Scale bar, 100 μm.

B Widespread expression of SLC6A20 β-gal fusion protein in various brain regions (P56), including the meninges, cortex, hippocampus, choroid plexus, and thalamus, as revealed by X-gal staining of brain sections from *Slc6a20a*$^{+/-}$ mice. Scale bar, 1 mm.

 © 2021 The Authors

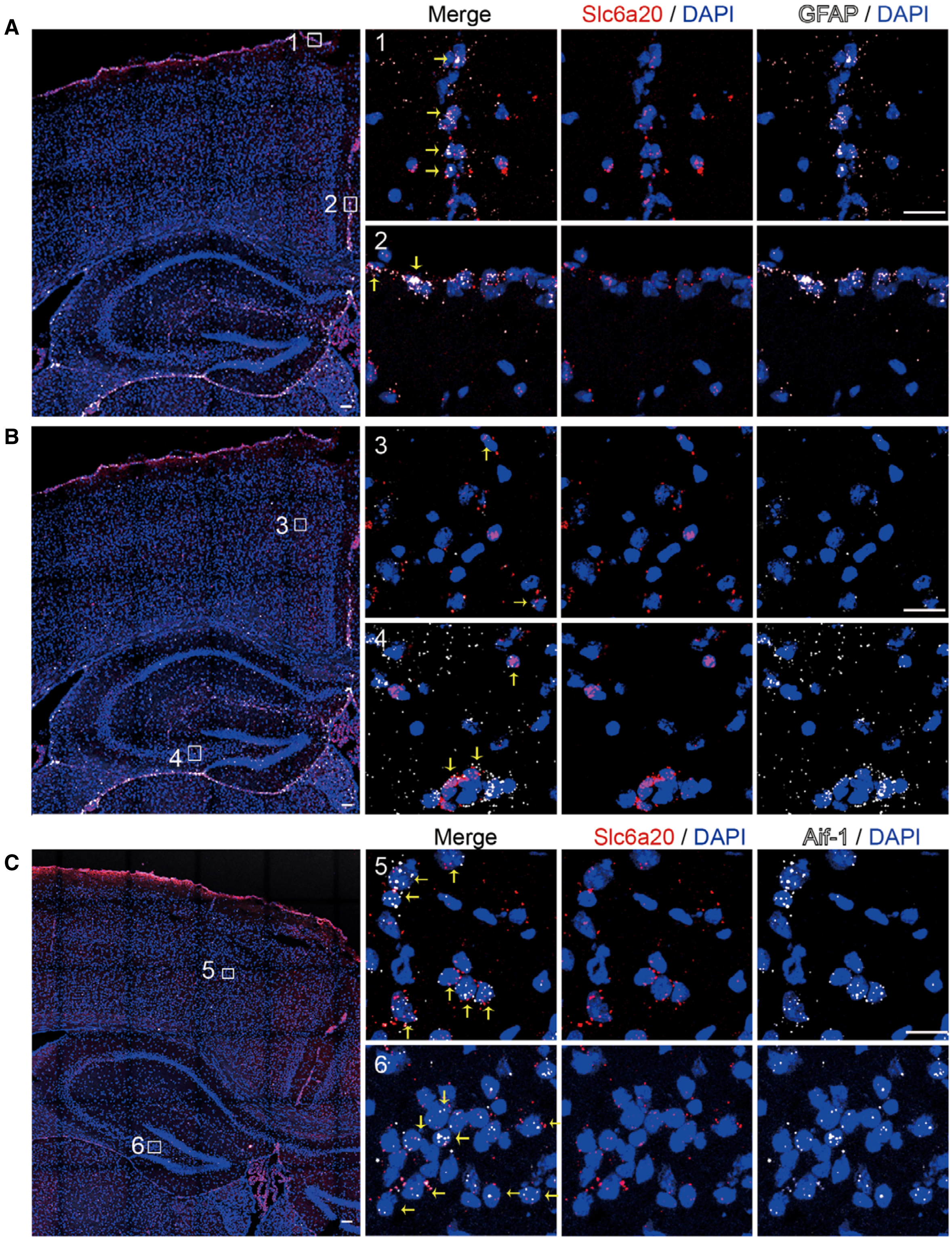

Figure EV4. Expression of *Slc6a20* mRNAs in meninges, astrocytes, and microglia.

A–C Expression of *Slc6a20* mRNA in meninges (A), astrocytes (B), and microglia (C) in the mouse brain (P56), revealed by double FISH. GFAP and Aif-1 were used as markers of astrocytes and microglia, respectively. Note that the panels A and B use the same original image to highlight different brain regions. Scale bar, 100 μm. Colocalization of Slc6a20 mRNA signals with cell markers are indicated by yellow arrows.

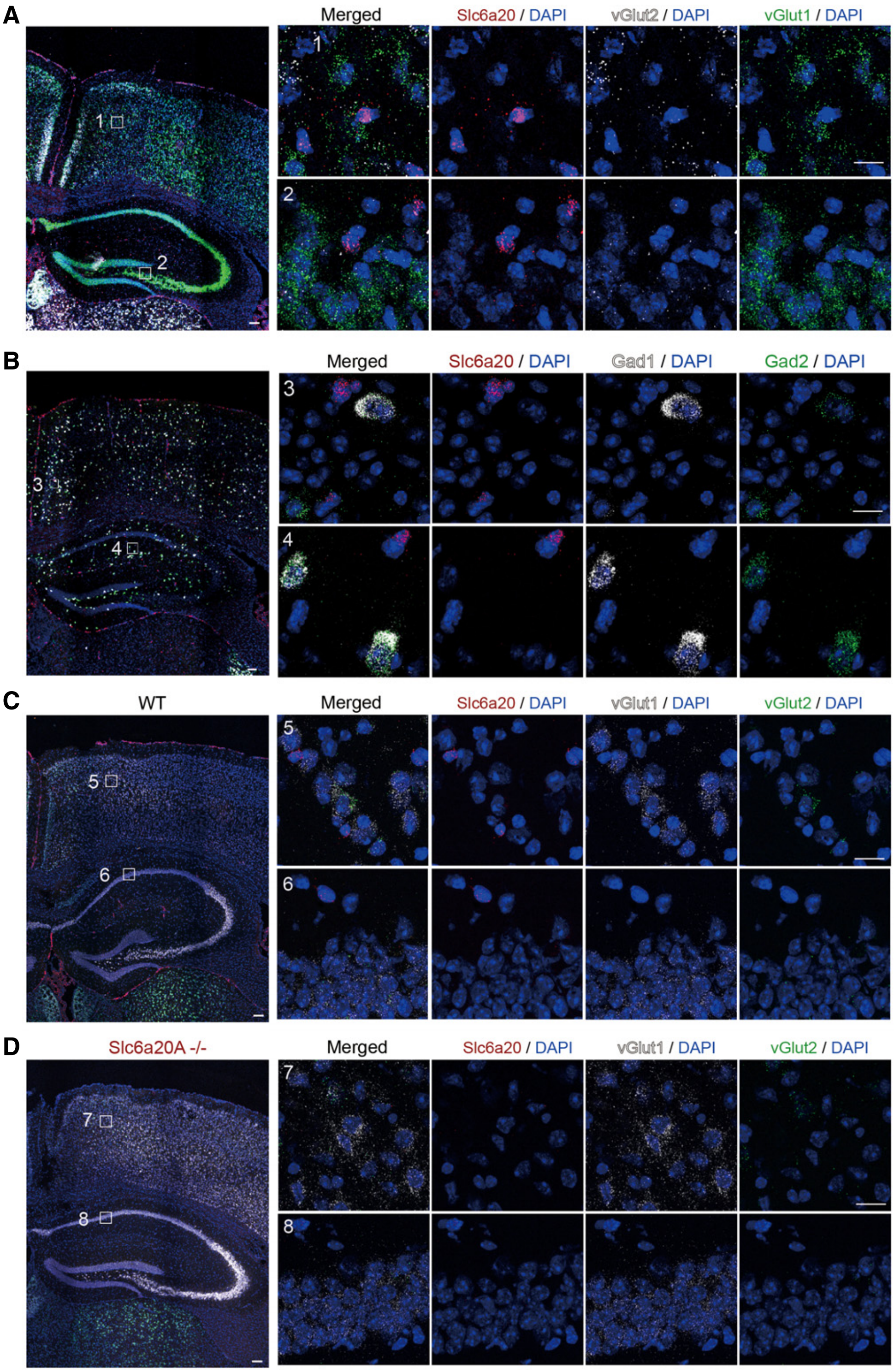

Figure EV5. Weak expression of Slc6a20 mRNAs in excitatory and inhibitory neurons.

A, B Modest and minimal expressions of *Slc6a20* mRNAs in *Vglut1*/*2*-positive glutamatergic neurons (A) and *Gad1*/*2*-positive GABAergic neurons (B), respectively, in the mouse brain (P56), revealed by double FISH. Scale bar, 100 μm.

C, D Demonstration of the specificity of *Slc6a20* mRNA signals by parallel double FISH experiments on WT (C) and *Slc6a20a*$^{-/-}$ (D) mice. Vglut1/2 and DAPI were used as controls. The residual *Slc6a20* mRNA signals in the panel (D), which are much weaker than those in the panel (C), may represent *Slc6a20b* (not *Slc6a20a*) mRNA signals. Scale bar, 100 μm.

 © 2021 The Authors
