# Supplementary figures and images for: SLC6A20 transporter: a novel regulator of brain glycine homeostasis and NMDAR function
Source: EMBO Mol Med. 2021 Jan 11;13(2):e12632. doi: 10.15252/emmm.202012632 (PMC7863395; doi:10.15252/emmm.202012632)

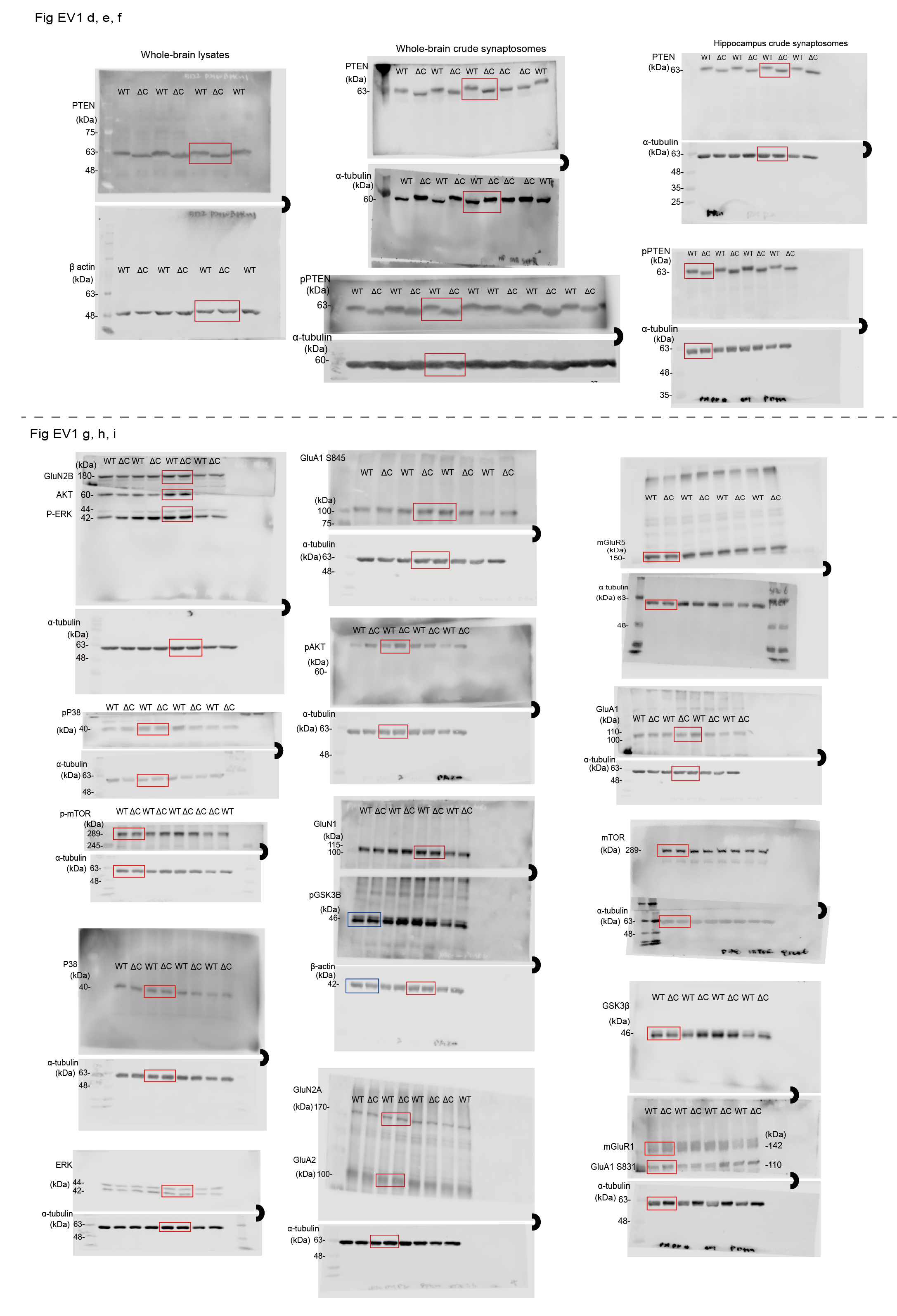

Supplement: Supplementary file 5 — Source Data for Expanded View [file EMMM-13-e12632-s009.zip › EMM-2020-12632-V3-Figure_EV1_Source_Data-sd.jpg]

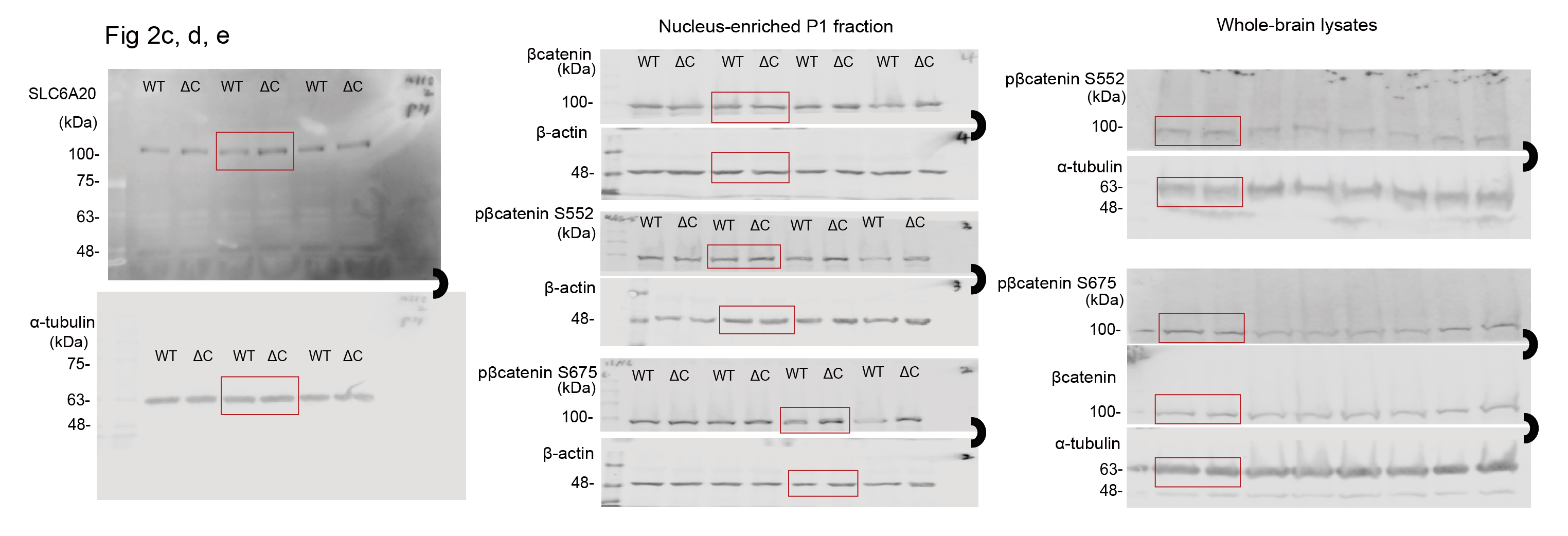

Supplement: Supplementary file 7 — Source Data for Figure 2 [file EMMM-13-e12632-s005.jpg]

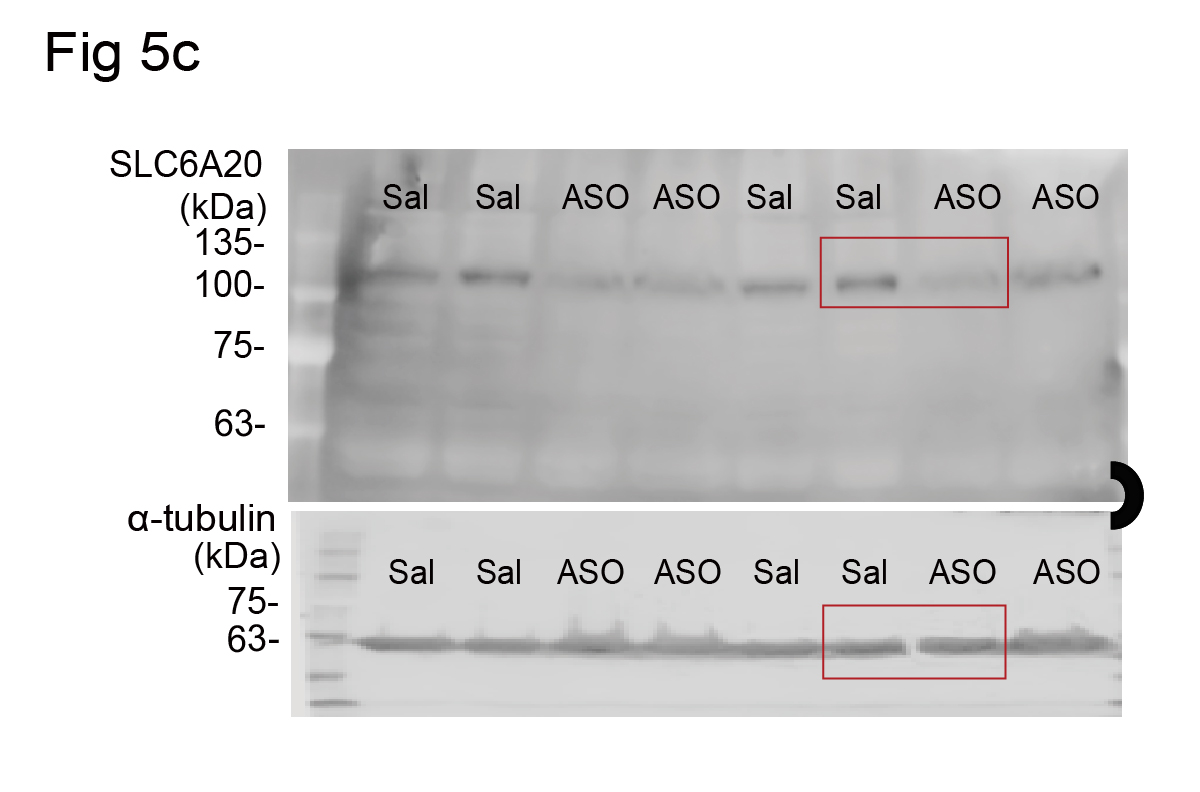

Supplement: Supplementary file 8 — Source Data for Figure 5 [file EMMM-13-e12632-s006.jpg]

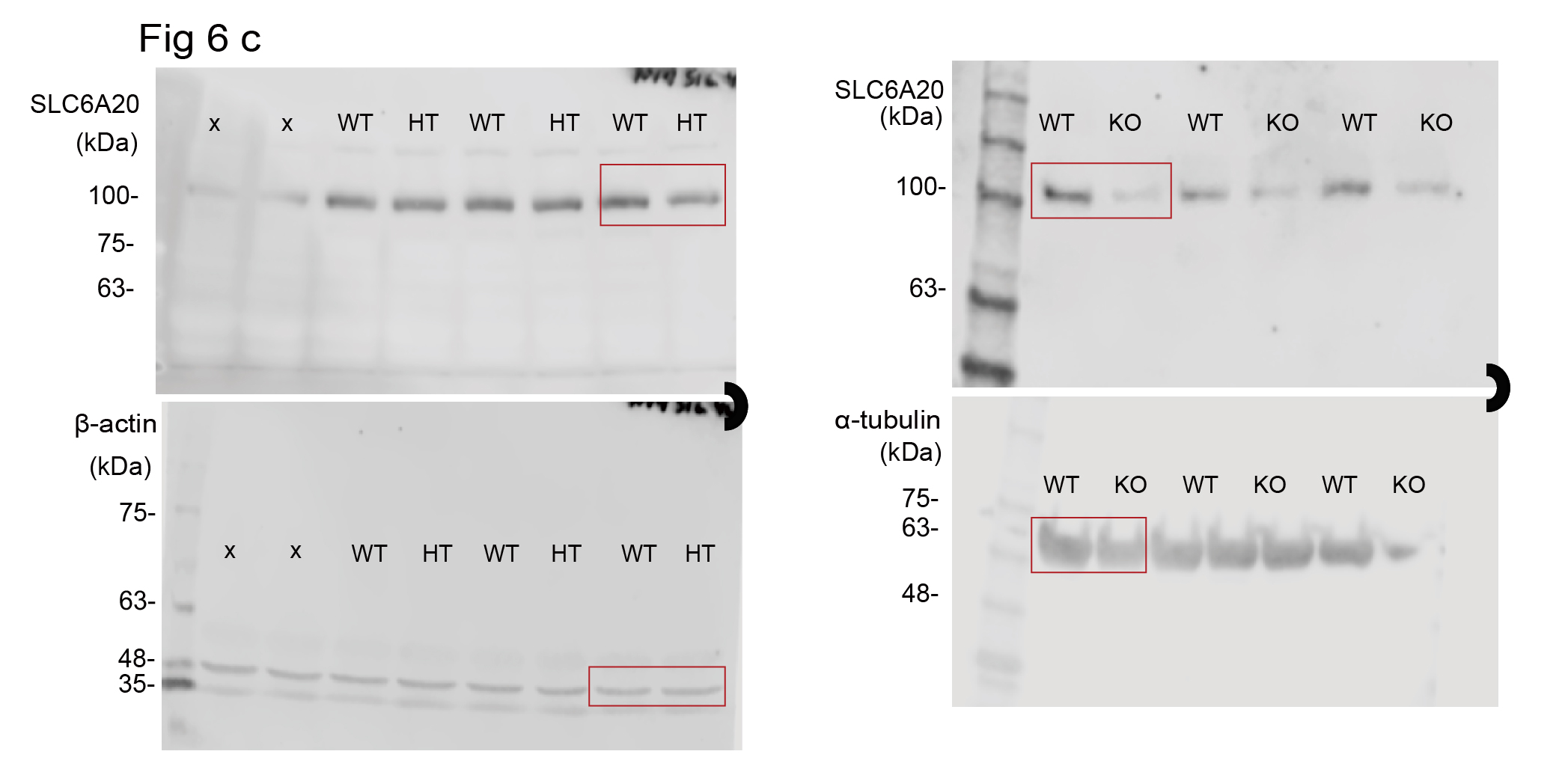

Supplement: Supplementary file 9 — Source Data for Figure 6 [file EMMM-13-e12632-s007.jpg]

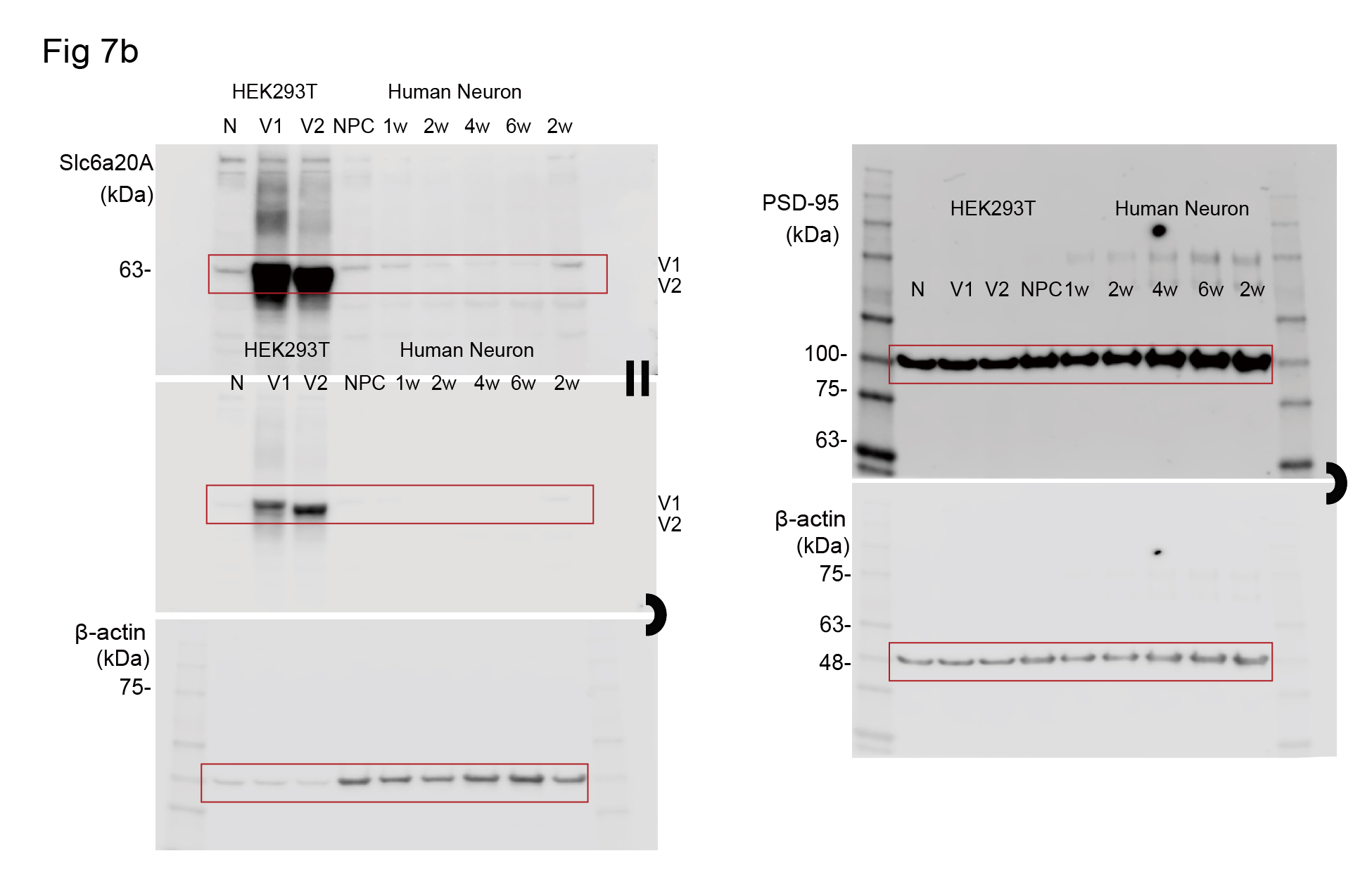

Supplement: Supplementary file 10 — Source Data for Figure 7 [file EMMM-13-e12632-s008.jpg]
